# The Impact of COVID-19 Confinement on Reading Behavior

**DOI:** 10.2174/17450179-v19-e230505-2022-42

**Published:** 2023-06-05

**Authors:** Mahmoud A. Alomari, Omar F. Khabour, Karem H. Alzoubi, Aseel Aburub

**Affiliations:** 1 Department of Rehabilitation Sciences, Jordan University of Science and Technology, Irbid, Jordan; 2 Department of Medical Laboratory Sciences, Jordan University of Science and Technology, Irbid, Jordan; 3 Department of Pharmacy Practice and Pharmacotherapeutics, University of Sharjah, Sharjah, UAE.; 4 Department of Clinical Pharmacy, Jordan University of Science and Technology, Irbid, Jordan.; 5 Department of Physiotherapy, Isra University, Amman, Jordan; 6 School of Health and Rehabilitation Sciences, Keele University, Newcastle Under Lyme, UK

**Keywords:** Reading behavior, COVID-19, Confinement, Pandemic, Lifestyle, Reading habits

## Abstract

**Background:**

The COVID-19 pandemic was detrimental to lifestyle and behavior. In this investigation, changes in reading habits during the pandemic were examined.

**Methods:**

The study is cross-sectional and survey-based. 1844 individuals completed an online survey about sociodemographic and reading habits during COVID-19. Multinomial logistic regression was used to examine the relationship between the study variables.

**Results:**

Most of the participants were active readers (71.5%-83.2%). Fewer (13.8-18.0%) reported a decrease in reading, while about half reported a no change, and 1/3^rd^ reported an increase. Changes in reading habits were related to age, education, job type, and income.

**Conclusion:**

About half of the participants during the pandemic reported a change in reading habits. Interventions to further enhance reading among people during the pandemic might help ameliorate the negative impacts of the pandemic.

## 1 INTRODUCTION

The SARS-CoV-2 that causes COVID-19 disease emerged in 2019, and in March 2020, the World Health Organization declared COVID-19 a pandemic [1], affecting most of the globe. As of January 2022, the virus has claimed over 330,000,000 cases and 5.560 million fatalities worldwide. All population segments are susceptible to COVID-19; however, the elderly with diseases seem most affected [2], and COVID-19 can be transmitted mainly by close contact with infected people or through contaminated objects [3].

After the World Health Organization declaration, many countries have resorted to different control strategies to intercept or slow the virus’s spread. These strategies included quarantine, curfew and lockdown, social distancing, sheltering at home, and complete isolation [4]. The pandemic and subsequent quarantine regulations might have affected people’s daily routines, lifestyles, and mental health [5]. Recently, it was shown that quarantine and staying at home during the COVID-19 pandemic increased anxiety, depression, stress, self-harm [6-8], and suicide attempts [9]. During the pandemic, reading literature was among the activities that remained available. Previous studies have suggested that limited outdoor activities during COVID-19-induced confinement were associated with increased alternative activities, namely reading [10-12]. During the COVID-19 pandemic, people started working from home, lost jobs, or turned to furlough [13]. These sudden changes may have spared more time for hobbies, leisure, and self-development, in which reading takes a major role.

Reading is the process of looking at a series of letters/symbols to acquire scientific, religious, entertainment, and general information. It is the cornerstone of self-enhancement and human development [14, 15]. It is associated with health and therapeutic benefits, including enhancing deep relaxation, self-esteem, inner calm, better sleep, and lowering stress and depression levels [16, 17]. It has been used for personality adjustment [18-20]. Factors that affect reading include mode of reading (electronic *vs*. hardcopy material) [21], gender [22, 23], age [24, 25], motivations [26], education qualifications and levels [25], and environment [27, 28].

Reading is one of the most common leisure activities in confinement [10-12]. For example, reading was ranked the number one preferred leisure time activity in long-duration space trips [10]. Additionally, books were the highest-ranked personal items that astronauts packed on long missions [10-12]. Furthermore, reading was one of the most practiced activities during leisure time in the Antarctic and submarine missions [12]. Moreover, prisoners have reported that reading helped them to improve peace of mind, enhance knowledge, strengthen character, and decrease the rate of recidivism [10-12].

During the COVID-19 pandemic, people were confined to homes and unable to attend to normal daily activities due to confinement regulations. Reading, including magazines, books, newspapers, or novels, might help reduce loneliness and improve mental health and well-being during “staying at home” [29-31]. On the other hand, libraries were closed during the pandemic, which might lead people to order book deliveries or to depend on electronic/audiobooks and magazines. Several famous publishers gave free access to electronic books to encourage people to cope with confinement during the pandemic [32]. However, it is not clear how quarantine and lifestyle changes affected the literary reading experience. In a study conducted in Algeria, about two-thirds of participants noticed changes in reading habits, and about 15% devoted 3 hours daily to reading books [30]. The current study aims to explore the changes in reading habits during COVID-19 confinement. We hypothesize that confinement during COVID-19 can favorably alter reading habits, allowing people to increase reading time. In addition, changes in reading habits during the pandemic are related to demographic factors. This study is essential to understand how confinement might change people’s behavior, specifically reading habits, and the sociodemographic factors (such as education, age, income, and employment) leading to these changes. Subsequently, the results can be used to develop interventions/strategies to enhance reading during the pandemic.

## 2 METHODS

### 2.1 Design and Participants

The data for the current study was obtained from the “Behavior, Knowledge, Stress and Quality of Life during COVID-19-induced Confinement (BKSQ-COVID19) project”, a cross-sectional study to examine changes in reading habits. An online survey was distributed among Jordanian adults during the second quartile of 2020. A snowball convenience sampling approach was used to anonymously recruit participants using social media platforms and applications, including Facebook groups, Instagram, WhatsApp groups, LinkedIn, Twitter, and institutional emails. Details about the study were provided, and electronic consent was obtained before the participants could access the survey. Institutional Review Board (IRB-JUST) approved the conduction of the study.

### 2.2 Questionnaire

Due to the uniqueness of the pandemic and applied confinement measures, no standard survey was proper to suit the study’s objectives. Thus, the research team developed a questionnaire based on similar studies [33]. Validation of content and face validation were done. At First, feedback was provided by a group of experts on the survey items. Their comments were implemented into the study survey. After that, pilot testing was carried out using the modified version of the study questionnaire, where participants (n=50) provided their opinion regarding the clarity and comprehensibility of the survey items. The responses from the pilot study were not included in the final data analysis. For all items of the study questionnaire, the test-retest reliability coefficient was ensured to be >0.65.

The study instrument collected information about the demographics of the participants (age, education, marital status, *etc*.), their views on Covid-19, and how the pandemic affected their reading habits. Additionally, the participants were asked to indicate the types of confinement procedures they experienced during the pandemic. The survey asked the participants four questions about the engagement level in reading specialized, general knowledge, story/novels, and holy books and material to determine the participants’ reading habits. The questions were: “What changes have you experienced in the following reading types due to the spread of COVID-19?”. Four choices were available, “increase,” “decrease,” “no change,” and “never practiced in this behavior.”

### 2.3 Data Analysis

The data were analyzed and presented as mean±SD, frequency, and percentages, using the SPSS (version 21) for statistical analysis. Multinomial logistic regression examined the relationship between age, gender, income, education, and job type with the reading indices. Reading indices include “Specialized books/journals,” “Holy books,” “General knowledge,” and “Stories/novels.” The Chi-square test was implemented to examine the differences in the participant responses to the reading questions, and the p-value was set at 0.05. Additionally, cross-tabulation was implemented to determine the association of the different demographic parameters (age, gender, income, education, and job type) with the responses of the participants to the survey items. Subsequently, cell percentages with adjusted standardized (AdRs) greater than ±1.96 were considered significantly different from the rest of the cells. The responses used in the statistical analysis were “increase,” “decrease,” and “no-changes.”

## 3 RESULTS

### 3.1 Participants

The demographics of the study participants are shown in Table **1**. The study instrument was filled by 1844 adult (18-72 years old) subjects. Most participants were female, held a university degree, and were employed. As in Table **2**, several confinement procedures were applied by the government, including self-quarantine, social distancing, school closing, and banning social gatherings (93.7-99%).

### 3.2 Prevalence of Reading Habits

Table **3** depicts that the majority of the participants reported that they were involved in reading habits. The results showed that 77.6%, 83.2%, 78.5%, and 71.5% of subjects reported reading specialized, holy, general knowledge, and story materials, respectively.

### 3.3 Changes in Reading Habits

Data analysis shows differences (*p*<0.001) in the responses to the reading habits question items, “increase,” “decrease,” versus “no-change.” Fig. (**1**) shows that ~50% (range: 47.7%-53.7%) of the participants reported a “no-change,” while a third reported an “increase” (range: 30.1-34.6) in reading habits.

### 3.4 Factors Contributing to the Changes in Reading Habits

Table **4** shows a multinomial regression of age, education, income, and job relationship with reading habits during the COVID-19 pandemic. Subsequent cross-tabulation showed that reading specialty material was related to age (ꭓ^2^ =12.3; p=0.01). Additional sub-analysis revealed that younger individuals reported the least (53.7%; AdRs=-3.2) “no-changes” and greatest (61.8%; AdRs=2.0) “increase” while the middle-aged (30-49 years old) experienced the greatest (34.6%; AdRs=2.8) “no-change” and least (25.5%; AdRs=-2.5). An association was also found between reading specialty material and education (ꭓ^2^ =19.8; p=0.003), with high school diploma holders experiencing the greatest (25.3%; AdRs=3.8) “decrease” while graduate diploma holders experiencing the least (12.7%; AdRs=-2.4) “decrease.” The cross-tabulation revealed a relationship (ꭓ^2^ =20.4.; *p*=0.0001) between reading specialty material with income. Additional comparisons showed that lower income was associated with the greatest (21.6%; AdRs=3.4) “decrease” and least (12.4%; AdRs=-2.3) “no-change” while higher income was associated with the least (4.1%; AdRs=-2.5) “decrease” and greatest (10.7%; AdRs=-2.5) “increase” and middle income associated with greatest (79.8%; AdRs=2.2) “no-change” in reading specialty material. The cross-tabulation test showed that reading specialty material was related to job type (ꭓ^2^ =31.4; p=0.01). Posthoc comparisons demonstrated that holding a job in education was associated with the greatest (29.6%; AdRs=2.0) “decrease” and a job in health was associated least (10.0%; AdRs=-2.6) with “decrease” and greatest “increase” (19.8%; AdRs=2.8). Additionally, a job in crafting was associated with the least (1.4%; AdRs=-2.3) “no-change,” while a job in engineering was associated with the greatest (7.7%; AdRs=3.3) “no-change” and least (3.5%; AdRs=-2.2) “increase.”

Reading holy books was related to participants’ education level (ꭓ^2^ =15.1; p=0.02). Further posthoc revealed that holding a high school diploma was associated with the greatest (24.5%; AdRs=2.0) “decrease” and least (17.0%; AdRs=-2.2) “no-change” while holding a graduate degree was related to the greatest (18.7%; AdRs=2.6) “no-change.” A relationship between reading holy books was found with income (ꭓ^2^ =10.2; p=0.04), with individuals in the low-income category experiencing the greatest (21.0%; AdRs=2.4) “decrease” and least (13.2%; AdRs=-2.4) “no-change.” A relationship between reading holy books was found with job type (ꭓ^2^ =29.3; p=0.02). Further post hoc tests showed that individuals holding a job in education experienced the greatest (33.7%; AdRs=-3.2) “decrease” and least (21.9%; AdRs=-2.2) “no-change.” Additionally, holding an engineering job was associated with most (13.2%; AdRs=-2.4) “no-change” as well as least (2.2%; AdRs=-2.1) “decrease” and (3.7%; AdRs=-2.0) “increase” in reading holy books.

Reading newspapers was associated with age (ꭓ^2^ =12.6; p=0.01). Subsequent posthoc analysis showed that younger individuals exhibited the greatest (61.3%; AdRs=-3.0) “increase” and least (53.2%; AdRs=2.3) “no-change,” while the elderly experienced the greatest (12.8%; AdRs=2.9) “no-change” and least (7.8%; AdRs=-2.3) “increase” in reading newspapers. A relationship (ꭓ^2^ =34.4; p=0.0001) was also found between reading newspapers and education. Subgroup comparisons revealed that individuals with a high school degree experienced the greatest (30.3%; AdRs=5.4) “increase” and least (16.2%; AdRs=-2.0) “no-change” and (15.3%; AdRs=-2.2) “increase.” Additionally, holding a graduate degree was associated with the least (10.4%; AdRs=-3.2) “decrease” and greatest (19.6%; AdRs=2.2) “increase” in newspaper reading. Reading newspapers was also associated with (ꭓ^2^ =13.4; p=0.009) income. The subsequent posthoc analysis revealed that low income was associated with the greatest (19.7%; AdRs=2.3) “decrease” and least (12.6%; AdRs=-2.3) “no-change.” Additionally, individuals with middle income reported the greatest (79.7%; AdRs=-2.3) “no-change,” while those with higher income experienced the greatest (4.0%; AdRs=-2.3) “increase” in newspaper readings.

Reading stories was associated (ꭓ^2^ =49.3; *p*=0.0001) with age. The younger individuals reported the least (50.9%; AdRs=-6.2) “no change” and the greatest (72.2%; AdRs=6.2) “increase.” Additionally, the middle-aged reported the greatest (36.4%; AdRs=4.4) “no-change” and least (23.3%; AdRs=-3.9) “increase” while the elderly experienced the greatest (12.7%; AdRs=3.4) “no-change” and least (4.5%; AdRs=-4.2) “increase” in reading stories. Furthermore, reading novels was related (ꭓ^2^ =33.7; *p*=0.0001) to education. Additional group comparisons showed that individuals with high school diplomas reported the greatest (28.7%; AdRs=4.6) “decrease” and least (15.4%; AdRs=-2.0) “no-change” while holding a two-year degree was associated with the least (36.4%; AdRs=-2.1) “decrease.” Additionally, individuals with a graduate degree reported the greatest (21.1%; AdRs=3.3) “no-change” and least (13.6%; AdRs=-2.5) “increase” while holding a bachelor’s degree was associated with the greatest (57.1%; AdRs=2.4) “increase” in reading stories. Changes in reading stories were also associated with (ꭓ^2^ =27.7; *p*=0.03) job type with holding a job in engineering was associated with the least (2.1%; AdRs=-2.2) “decrease” and greatest (7.4%; AdRs=3.3) “increase.”

## 4 DISCUSSION

COVID-19 has compelled governments around the globe to impose a variety of confinement measures [4] that are usually associated with an evident increase in stress and crucial lifestyle changes [6-8]. However, the effect of COVID-19-induced confinement on reading habits is not well investigated. Therefore, the current study examined the changes in reading habits during the COVID-19 pandemic.

Due to the pandemic, the confinement measures were self-quarantine, physical distancing, banning group events, school closing, and lockdowns. According to the results, most participants were also active readers (71.5%-83.2%). Less (13.8-18.0%) percent reported a decrease in reading, about half reported a no change, and 1/3^rd^ reported an increase. Importantly, changes in the reading specialty material were related to age, education, job type, and income, while education, income, and job type were associated with changes in reading holy books. Furthermore, changes in reading newspapers were related to age, education, and income, while changes in reading stories were associated with age, education, and job type. The results agreed with a study conducted in Algeria which reported changes in reading habits in most participants attributed to COVID-19 pandemic confinement measures [30]. In that study, about 15% of participants read more than 3 hours daily [30].

Confinements are rarely experienced in situations such as imprisonment, Antarctic camps, and space trips. Consistent with studies examining the effect of confinement on reading habits [10-12], all reading types increased among the majority while some decreased during the COVID-19 lockdown period. Previous reports showed increased reading time [10-12] during confinement, including imprisonment, the South Pole, and airspace trips. Tamilmani (2014) explained that books are the voices of people living in confinement, especially in quiet environments [34].

Fortunately, the current study findings are advantageous for mental health. Several studies have reported increased mental health distress during the COVID-19 lockdown period [6-8]. Previous data have indicated that people who read more during confinement experienced fewer mental health disorders, including depression, anxiety, and stress [17]. Accordingly, people are recommended to fulfill free time during confinement by embracing rewarding hobbies and enjoyable activities, such as reading. Additionally, strategies should be implemented to encourage people to read during long-term confinement for self-development and diverting confinement-induced stress [10-12]. Alternatively, the advantages of reading extend beyond leisure and psychological benefits. Reading was suggested among the solutions to deal with sleeping problems by the European CBT‐I Academy [16]. Additionally, reading is essential for training the imagination, exercising the brain, expanding knowledge, enhancing literacy, and improving learning outcomes [35]. Studies have shown that reading can improve social skills [36], health [37], and overall quality of life [38].

This is the first study that addresses factors associated with reading habits during COVID-19-induced confinement. Consistent with previous literature in ordinary circumstances [21], changes in reading in the current study were related to demographics (*i.e*., age and gender) and socioeconomic status (*i.e*., education and income). For example, reading newspapers correlated with gender and education and, to a greater extent, age [39]. Additionally, Chokron *et al*. (2000) reported that cultural factors, including perception about the importance of reading, implementing extra-curriculum reading from childhood, and reading advisements in the media, were detrimental to reading under normal circumstances [40]. Similarly, McGeown *et al*. (2015) identified gender and age as the main factors that affect reading habits [22]. Moreover, Raghunandan *et al*. (1996) clustered factors affecting reading into four groups: ecological (the various environments; emotional (the social and psychological factors); physical (vision, hearing, age, gender, and other health factors), and educational factors [23]. Additionally, in a recent systematic review, the internet (speed, downloading limit, and having accounts on social media), environment (home environment,) and motivation were reported as the main factors affecting reading [26].

The current study is a novel study that explored reading habits during the COVID-19 confinement period. The results might help make plans and implement strategies to further enhance reading. For example, knowing the most desired reading material might help to produce more suitable books/articles. Subsequently, accommodate different population segments, including genders, age groups, and education and income levels. However, more studies are needed to understand the importance of reading for health, particularly mental and psychological health, during disease and long-term confinement.

This study is not without limitations. These limitations include; the study design was cross-sectional, the number and scope of the reading questions were sparse, and it did not assess the effect of reading on mental and psychological status. Therefore, future longitudinal and interventional studies are warranted to survey in more detail the people’s favorite reading topics and types (*i.e*., e-books, hard copy books, audiobooks, *etc*.). Additionally, more studies are warranted to explore the health, particularly mental and psychological, benefits of reading during long-term disease-induced confinement. Another possible limitation is the possibility of selection bias, where it is possible that more educated than the general population have responded to the survey. However, the educational levels in the current study sample were comparable to the nationwide statistics shown by the Jordan Department of Statistics. Thus, it is unlikely that more educated people only responded to this survey. Finally, online surveys can suffer from multiple enrollments in the same subject. However, such a possibility is also not likely as most study subjects will only be willing to complete the data survey only once.

## CONCLUSION

Most participants reported either a “no-change” or an “increase” in reading habits. Additionally, the data show that age, education, income, and job type predict reading habit changes. These results suggest that the COVID-19-induced confinement might impose a no-change or positive change in reading. However, studies and interventions are warranted to further understand changes in reading and to design and implement strategies to encourage reading during long-term disease-induced confinement.

## Figures and Tables

**Fig. (1):**
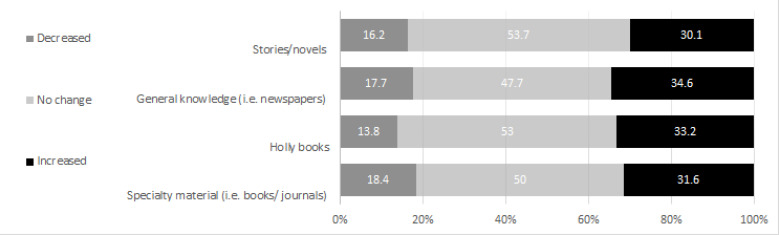
Prevalence of changes in reading habits (%).

**Table 1:** Demographics of the study sample (n=1844).

**Males (%)**					30.5			
**Females (%)**					69.5			
**Age (mean±SD)**					33.7 ± 11.3			
**Education (%)**					-			
-	≤ High school				19.4			
-	Community college				14.1			
-	University (Bachelor)				51.3			
-	High education (MSc/PhD)				15.3			
**Family income (%)**					-			
-	Low income				34.5			
-	Middle income				65.5			
-	High income				-			
**Employment status (%)**					-			
-	Unemployed/retired				35.6			
-	Employed				64.5			

**Table 2:** Reported confinements during COVID-19 (n=1844).

**Infection likelihood**						-			
-		Low				59.5			
-		Moderate				34.5			
-		High				6.0			
**Knowing a COVID-19 patient**						-			
-		Yes				6.3			
-		No				93.7			
**Did you Self-quarantine**						-			
-		Yes				93.5			
-		No				6.5			
**Social distancing**						-			
-		Yes				96.8			
-		No				3.2			
**Group events were banned (*e.g*., weddings)**						-			
-		Yes				98.2			
-		No				1.8			
**Closure of schools**						-			
-		Yes				99.0			
-		No				1.0			
**Curfew**						-			
-		Yes				97.0			
-		No				3.0			

**Table 3:** Prevalence of various reading types.

**Type of Reading**							**Participation**				**No Participation**	
Specialized books/ journals (%)							77.6				22.4	
Holy books (%)							83.2				16.8	
General knowledge (*i.e*. newspapers) (%)							78.5				21.5	
Stories/novels (%)							71.5				28.5	
Values presented are the percent (%)												

**Table 4:** Multinomial regression between confounding factors and types of reading.

-			**Specialized**	**Holly**				**General Knowledge**				**Story**	
Age			**ꭓ^2^=11.7; *p*=0.02**	ꭓ^2^=9.6; *p*=0.5				ꭓ^2^=12.2; *p*=0.2				**ꭓ^2^= 42.2; *p*= 0.000**	
Education			ꭓ^2^=11.5; *p*= 0.07	ꭓ^2^=9.0; *p*=0.2				**ꭓ^2^=21.8; *p*= 0.002**				**ꭓ^2^= 28.2; *p*= 0.000**	
Income			**ꭓ^2^=11.9; *p*= 0.02**	ꭓ^2^=5.8; *p*=0.2				ꭓ^2^=8.8; *p*= 0.06				**ꭓ^2^= 10.4; *p*= 0.03**	
Job type			ꭓ^2^=4.2; *p*=0.4	**ꭓ^2^=9.8; *p*=0.04**				ꭓ^2^=2.5; *p*= 0.7				ꭓ^2^= 5.1; *p*= 0.3	

## Data Availability

The data supporting the findings of the article is available upon request *via* e-mail to the corresponding [K.H.A] author.

## LIST OF ABBREVIATIONS

AdRs: = Adjusted Standardized
COVID-19: = Corona Virus 19
